# Mind Your Heart—A Web-Based Dietary Approaches to Stop Hypertension Diet and Mindful Eating Intervention for Working Adults at Elevated Cardiovascular Risk: Pilot Randomized Controlled Trial

**DOI:** 10.2196/106388

**Published:** 2026-10-28

**Authors:** Anita M Reina, Heather M. Padilla, Jenay M Beer, Donglan S Zhang, Lisa M. Renzi-Hammond

**Affiliations:** 1 Wellstar College of Health & Human Services, Department of Health Promotion and Physical Activity, Kennesaw State University, 590 Cobb Ave. NW, Room 3049, Convocation Center, Kennesaw, GA, 30144, United States, 1 470-578-6216, 1 470-578-9031; 2 College of Public Health, Institute of Gerontology, University of Georgia, Athens, GA, United States; 3 College of Public Health, Department of Health Promotion and Behavior, University of Georgia, Athens, GA, United States; 4 Division of Health Services Research, Department of Foundations of Medicine, New York University Long Island School of Medicine, Mineola, NY, United States

**Keywords:** DASH Diet, mindful eating, diet adherence, diet behaviors, eHealth, Dietary Approaches to Stop Hypertension Diet

## Abstract

**Background:**

The Dietary Approaches to Stop Hypertension Diet (DASH) diet supports heart health; yet, many find it difficult to follow in community settings. Mindful eating can improve adherence, but in-person programs are time-consuming and not always accessible. eHealth offers an accessible, scalable alternative that may increase engagement and compliance.

**Objective:**

This study assessed the feasibility of a DASH eHealth program focused on mindful eating and the preliminary changes in eating behaviors, diet quality, and health outcomes.

**Methods:**

Participants were recruited from a university workplace via email and flyers and randomly assigned to either a web-based DASH diet education program (Diet Only) or the Mind Your Heart (MYH) intervention. The MYH program included the same DASH curriculum plus additional mindful eating instruction, guided practice, and specific, measurable, achievable, realistic, and time frame (SMART) goal setting. Both interventions were fully asynchronous, self-guided, and lasted 6 weeks on the Desire2Learn (D2L) Brightspace platform, with no personalized coaching or counseling. Baseline and postintervention assessments involved in-person measurements of anthropometrics, blood pressure, and blood biomarkers. Participants recorded their diets electronically over 3 days using MyFitnessPal (MyFitnessPal, Inc) and completed validated questionnaires on mindful eating using the Mindful Eating Behaviors Scale (MEBS), eating behaviors using the Three-Factor Eating Questionnaire Revised 21-item (TFEQ-R21), motivation for regulated eating using the Regulated Eating Behaviors Scale (REBS), and perceived stress using the Perceived Stress Scale 10-item (PSS-10). During the intervention, automated ecological momentary assessments (EMAs) were sent electronically 3 times weekly to monitor eating behaviors in real time.

**Results:**

Thirty-seven participants (mean BMI 31.93 SD 7.25, 34/37, 91.9% female, 31/37, 83.8% White) were randomized into groups. Among the 36 who remained enrolled, the mean curriculum completion was 85.9%, SD 21.4; 31 (86.1%) completed≥60% of assigned content, and 32 (88.9%) completed at least half of the scheduled EMA prompts. Thirty-four participants attended the postintervention assessment and completed the questionnaires and the blood pressure assessment, although paired baseline and postintervention data were not available for all anthropometric measures and biomarkers. Participants reported no adverse events, and research staff observed none. Although no significant between-group differences were observed for DASH adherence, anthropometric measures, blood pressure, or cardiometabolic biomarkers, participants in the MYH group showed greater improvements than the Diet Only group in TFEQ-cognitive restraint (mean difference in change 0.39, 95% CI 0.06-0.72; Hedges *g*=0.81; *P*=.02) and REBS-controlled regulation (mean difference in change 3.59, 95% CI 0.01-7.16; Hedges *g*=0.68; *P*=.05).

**Conclusions:**

A fully asynchronous digital mindful eating plus DASH education intervention and associated research procedures were feasible among working adults at elevated cardiovascular risk. Preliminary changes in eating behaviors suggest that mindful eating warrants further evaluation, but findings should be interpreted cautiously given the small sample and feasibility design. Larger, adequately powered randomized controlled trials are needed.

## Introduction

Unhealthy diets characterized by the overconsumption of ultraprocessed foods, excess sodium, and low intake of fruits, vegetables, and complex carbohydrates account for about 11 million total deaths and about 255 million disability-adjusted life years lost worldwide [1]. High dietary sodium consumption alone is responsible for approximately 3 million deaths annually and results in about 70 million disability-adjusted life years lost, predominantly due to cardiovascular diseases (CVDs), irrespective of age, sex, or other socioeconomic variables [1]. Dietary modification is essential to primary and secondary prevention strategies for hypertension, the leading cause of CVD [2,3]. One evidence-based approach to dietary modification is the Dietary Approaches to Stop Hypertension (DASH) diet, which supports cardiovascular health by restricting saturated fat, cholesterol, and sodium intake and encouraging increased intake of calcium, magnesium, potassium, and fiber [4]. Clinical trials and epidemiological studies demonstrated that the DASH diet significantly reduces blood pressure [5-8]. Additionally, the DASH diet reduces other CVD risk factors, including lowering total cholesterol, fasting glucose, and BMI [9-13].

Despite these health benefits, adherence to the DASH diet is often poor outside controlled feeding trials [14]. Therefore, further research on adherence to diets like the DASH diet is necessary. A recent meta-analysis showed that even minor, incremental improvements in adherence to the DASH diet are associated with a clinically significant 5% reduction in all-cause mortality and approximately a 3% reduction in cardiovascular mortality [8]. Behavioral supports that aim to enhance adherence to high-quality diets, such as the DASH diet, in real-world settings are necessary to realize their therapeutic effects.

Mindful eating, a practice grounded in mindfulness principles, emphasizes focused awareness and nonjudgmental attention to the physical and mental experience associated with food choices and the eating environment [15]. It has shown promise in enhancing self-regulation of dietary behaviors, which are essential for managing CVD risk factors like diet and blood pressure [16,17]. The Mindfulness-Based Blood Pressure (MB-BP) Reduction framework emphasizes how attention control, emotional regulation, and self-awareness affect CVD risk factors, with studies [18-20] showing improvements in blood pressure, DASH diet adherence, and physical activity in mindfulness intervention groups compared with standard care. Despite these benefits, existing mindful eating and MB-BP interventions have been delivered almost exclusively in person, requiring substantial time, facilitator involvement, and participant commitment. These intensive formats limit scalability and access for many adults who could benefit.

Exploring digital, eHealth-based delivery offers a flexible, scalable way to provide mindful eating training, boost user engagement, and improve adherence to medical therapies, such as reducing the risk of CVD and stroke [21]. Broadly defined as health services delivered or enhanced through digital technologies [22-24], eHealth platforms provide a flexible, asynchronous, and multimodal content that accommodates busy schedules and learning preferences. However, sustained user engagement remains a persistent challenge, and many individuals disengage over time [25]. While prior research has largely focused on factors influencing initial adoption, such as perceived usefulness and ease of use [26-28], long-term adherence depends on more dynamic design features, including interactive prompts, real-time feedback, and ongoing digital touchpoints that reinforce behavior and support accountability. Ecological momentary assessment (EMA) is one strategy for enabling real-time behavioral monitoring and user engagement. By capturing eating behaviors and contextual factors in real-time, EMA provides insights into environmental triggers that influence dietary choices [29]. Text message-based EMA prompts yield higher response rates than paper-and-pencil prompts [30], although careful consideration of participant burden is necessary to ensure feasibility in dietary studies [29].

Mindful eating interventions may enhance dietary self-regulation and reduce CVD risk, yet traditional in-person delivery formats are resource-intensive and limit accessibility. Delivering mindful eating through eHealth platforms offers a potentially scalable alternative, but its feasibility in this context remains unclear. Although researchers have evaluated digital dietary education and mindfulness-based approaches independently and in other multicomponent interventions, less is known about the feasibility of integrating asynchronous DASH education with structured mindful-eating instruction and practice while also implementing intensive behavioral and physiological assessment procedures. Before conducting a larger trial, it was important to determine whether participants would engage with and complete the combined digital intervention and whether repeated EMA, dietary recording, behavioral assessments, and cardiometabolic measurements could be implemented with adequate retention and data completion. This study therefore evaluated the feasibility of the Mind Your Heart (MYH) intervention and study procedures while exploring preliminary differences in dietary, behavioral, and health outcomes between MYH and an active DASH education comparison condition. We hypothesized that participants would demonstrate high engagement and completion rates, with preliminary patterns suggesting improvements in eating behaviors, dietary adherence, and related physical health outcomes.

## Methods

### Study Design

This 6-week, 2-arm, parallel-group, subject-masked, randomized feasibility trial evaluated participant engagement with the full MYH eHealth curriculum and examined preliminary effects on DASH adherence, diet quality, mindful eating behaviors, and health outcomes. Participants were randomized in a 1:1 allocation ratio to one of two 6-week asynchronous eHealth programs delivered through the same online e-learning platform (1) a Diet Only comparison group that received DASH diet education, or (2) the MYH mindful eating group that received the same DASH diet education plus additional mindful eating training and guided mindfulness exercises. Exploratory outcome analyses compared pre- to postintervention changes between the MYH and Diet Only groups.

### Ethical Considerations

The study protocol, including the study design, eligibility criteria, intervention procedures, outcome measures, and planned analyses, was developed a priori and approved by the Sterling Institutional Review Board (IRB; PROJECT00003799) before participant recruitment. All participants provided written informed consent before completing any study procedures. Participants were informed that participation was voluntary, that they could withdraw at any time without penalty, and that their data would be handled confidentially using unique study identification numbers. Electronic data were stored on secure university systems, and identifiable information was retained only as long as necessary to administer the study before being removed. The study was conducted in accordance with the ethical principles of the Declaration of Helsinki.

### Trial Registration

Participant recruitment and enrollment occurred from January through March 2022, and data collection was completed in May 2022. The trial was not prospectively registered with ClinicalTrials.gov because the investigators did not recognize the requirement to register a clinical trial before participant enrollment. To promote transparency and public accessibility of the study protocol and prespecified outcomes, the trial was retrospectively registered before publication (ClinicalTrials.gov Identifier NCT07670910; 06-26-2026). The IRB-approved study protocol established the study objectives, primary and secondary outcomes, eligibility criteria, and planned analytical approach before participant enrollment. Retrospective trial registration did not result in changes to the study objectives, outcomes, or eligibility criteria after study initiation. Although the planned statistical methods were specified before analysis, the criteria used to determine the final analytic sample were made after data collection and are described transparently in the Data Analysis section. Future fully powered efficacy trials of this intervention will be prospectively registered. Multimedia Appendix 1 includes a comparison of the original study protocol, submitted paper, and revised analytic approach.

### Participant Recruitment

The study took place at the University of Georgia worksite with about 10,000 employees, mostly female, matching the final sample. Many working adults with or at risk for hypertension can participate in workplace health programs. Therefore, this feasibility study was carried out at a single site in the “stroke belt,” a region noted for high rates of hypertension, CVD, and stroke. Recruitment efforts included email invitations distributed through university staff listservs, accompanied by electronic flyers containing a web link and a QR code that directed recipients to an online eligibility screener. Department managers and wellness advocates also distributed the recruitment materials to full-time staff to promote participation. Participants were given a US $20 check for attending both data collection sessions. Although they received a US $20 incentive for completing assessment visits, they received no additional rewards for finishing modules. Therefore, curriculum engagement likely reflects genuine adherence rather than incentive-driven behavior.

### Study Sample and Allocation

Inclusion criteria included full-time employment, English fluency, and access to a computer and a smartphone with internet access. Exclusions were pregnancy or plans within the past 3 months, specialized diets for chronic illness, weight-loss medications, or enrollment in other dietary or mindfulness programs.

Because greater mindfulness is associated with carrying out health behaviors, eligible participants who consented and enrolled in the trial completed the Mindful Attention Awareness Scale (MAAS) [31] during the initial preintervention intake. To control for individual differences in baseline trait mindfulness, we stratified participants into relatively high- or low-level trait mindfulness based on MAAS scores using a procedure described by Alatiq [32]. Because participants enrolled on a rolling basis, we randomized periodically as participants completed baseline assessments. We first stratified participants into relatively high- and low-trait mindfulness groups based on MAAS scores. Within each stratum, we used a computer-generated random allocation sequence to assign participants to the Diet Only or MYH group in an approximately 1:1 ratio. To maintain balanced group sizes during rolling enrollment, the principal investigator occasionally made minor allocation adjustments. We assigned coworkers and couples who enrolled together to the same study condition to minimize the potential for intervention contamination. For each linked pair, we randomized the participant who enrolled first using the standard randomization procedure, and the partner or coworker was subsequently enrolled in the same condition. We used this procedure for 1 couple and 1 coworker pair.

The principal investigator generated the computer-based random allocation sequence, enrolled participants, and assigned participants to study groups using the previously described stratified randomization procedure. Because this was a small pilot feasibility trial with rolling enrollment, we did not use allocation concealment procedures (eg, centralized randomization or sequentially numbered opaque sealed envelopes). A research assistant then enrolled participants in their assigned Desire2Learn (D2L) course and informed them about the study procedures. Participants and the principal investigator were not blinded to intervention assignment. Participants were informed that they would be assigned to 1 of 2 web-based nutrition education programs but were not informed that 1 program included a mindful eating component, which condition represented the intervention of interest, or the study hypotheses. Participants learned about the content of their assigned program only after beginning the intervention.

As part of the feasibility study, we did not conduct a formal power calculation. The target sample size (n≈30‐40) follows guidelines for pilot and feasibility studies to estimate parameters like recruitment, retention, and variance for future trials [33].

### MYH Curriculum

The MYH program was developed to provide evidence-based DASH diet and mindful eating education tailored to working adults at elevated cardiovascular risk. The study team developed all educational content from evidence-based clinical guidelines and reviewed it before implementation to ensure scientific accuracy and consistency. Before implementing the intervention in the current randomized feasibility trial, we evaluated it in a previously published one-arm feasibility study involving a similar population [34]. Findings from that study informed refinement of the curriculum, usability, and overall program design [34]. Dietary content followed guidelines from the DASH diet [4], the Dietary Guidelines for Americans [35], and the American Heart Association [36]. In addition to translating biomedical CVD terminology into layperson’s terms, key topics included balanced eating, portion control, and reading nutrition labels.

The MYH mindful eating component emphasized attention control, emotional regulation, and self-awareness using intervention mapping and the MB-BP framework factors to support dietary self-regulation and reduce CVD risk [18,37,38]. The intervention drew on established mindfulness-based frameworks, including the MB-BP framework [18], and adapted guided mindful eating exercises from established mindfulness frameworks [39-41], but was not based on a single standardized, manualized program. The study investigators developed and recorded the mindful eating content, including a team of health promotion researchers and a health professional with a Master of Science in Nutrition, more than 20 years of experience in health education and behavior change, and an experienced registered yoga teacher at the 500 hour level (E-RYT 500) certification as an experienced registered yoga teacher. The intervention was delivered through the D2L Brightspace learning management system.

Both groups received 6 weekly asynchronous DASH-focused modules delivered through D2L Brightspace. One module was released each week and remained open throughout the intervention, allowing participants to review previous information. The core DASH modules were designed to take approximately 20‐30 minutes per week to complete and included instructional videos with embedded “test your knowledge” activities, forums for setting personalized SMART (specific, measurable, achievable, realistic, and time frame) goals, and weekly reflection activities. The DASH curriculum included approximately 112 minutes of audiovisual content across the 6-week intervention (mean 18:38 minutes/week [SD 6:55] ). Participants in the MYH condition received the same core DASH curriculum plus approximately 88 minutes of additional mindful-eating instructional and guided-practice audiovisual content (mean 14:42 minutes/week [SD 4:23]), as well as mindful-eating reflection, SMART goal-setting, and independent practice activities that were not available to the Diet Only condition. The intervention provided standardized content to all participants and did not include individualized tailoring, adaptive algorithms, or automated personalized feedback. Audiovisual durations do not include time spent reading written materials, completing interactive knowledge checks, reflections and goal-setting activities, or engaging in independent practice. The intervention content was finalized before participant enrollment and remained unchanged throughout the study.

The intervention was designed to be fully asynchronous and self-directed. Participants did not receive individualized coaching, counseling, or clinician support during the intervention. We sent standardized weekly reminder emails to encourage module completion, and research staff were available to provide technical assistance if participants had difficulty accessing the D2L Brightspace platform or completing study procedures. Technical support was provided upon participant request and did not include behavioral counseling or personalized dietary guidance.

Multimedia Appendix 2 provides representative screenshots of the eHealth program on D2L. Multimedia Appendices 3 and 4 provide a detailed module-by-module description of the intervention content, participant activities, and delivery for DASH education and mindfulness additions.

### Feasibility Measures

The reported feasibility indicators encompass recruitment, retention, completion of data collection, and EMA responsiveness. Recruitment metrics evaluated the proportion of individuals who screened, satisfied eligibility criteria, and ultimately enrolled in the study, reflecting the practicality of identifying and engaging the target population. Retention rates indicated whether participants remained enrolled throughout the 6-week intervention and attended both assessment visits, providing information about continued participation and the feasibility of study procedures. Although adverse events were not assessed using a formal structured monitoring protocol, research staff monitored for adverse events during in-person study procedures (eg, blood draw complications, dizziness, and fainting, etc), and participants could report concerns or adverse experiences to the research team throughout the study. The study team documented any adverse events reported by participants or observed by research staff.

Data collection completion rates, including anthropometrics, blood pressure, blood biomarkers, dietary records, and questionnaires, assessed participants’ willingness and ability to complete the assessments in both in-person and self-reported formats. Engagement with MYH programming was calculated as the number of education modules each participant completed on the D2L platform, as determined by group assignments. The platform provided data on module visits and engagement time (in hours and minutes). D2L-generated time-spent data were recorded for each participant by module; module-level times were summed across the 6-week intervention and averaged across participants. Because D2L generated these values, we could not determine whether periods of inactivity while content remained open were included. Finally, EMA responsiveness, defined as the proportion of text-message prompts completed during the intervention, served as an additional feasibility indicator and reflected participants’ engagement with more frequent, real-time data collection procedures.

### Behavioral Measures

#### Mindful Eating Behaviors

Mindful eating behaviors were measured using the Mindful Eating Behaviors Scale (MEBS) and the Three-Factor Eating Questionnaire (TEFQ-R21). The MEBS [17] is a 17-item tool that measures mindful eating behaviors. Although a total MEBS score is not computed, the MEBS includes 4 domains: focused eating, hunger and satiety cues, eating with awareness, and eating without distraction*.* Higher scores indicate greater mindfulness in that domain. The TFEQ-R21 [42,43] is a 21-item scale designed to measure 3 domains of eating behavior: cognitive restraint, uncontrolled eating, and emotional eating*.* Higher scores indicate greater levels of the respective factors.

#### Motivation for Regulated Eating

The Regulated Eating Behaviors Scale (REBS) [44] contains 24 items measuring 6 motivation levels for regulated eating behaviors: intrinsic, integrated, identified, introjected, extrinsic, and amotivation. These subscales form global scores for 2 types of regulation: autonomous and controlled. Autonomous regulation includes intrinsic, integrated, and identified motivation, reflecting internal reasons for healthy eating. Controlled regulation includes introjection, extrinsic motivation, and amotivation, driven by external factors such as others’ perceptions and shame. Both scores range from 12 to 84, with higher scores indicating stronger motivation. A total score combines these, weighted by their place on the self-determination continuum: positive weights (+3,+2,and +1) for autonomous, negative weights (–1,‐2, and −3) for controlled regulations, then summed.

#### Stress

The Perceived Stress Scale (PSS-10) [45,46] is a 10-item questionnaire designed to assess how individuals perceive situations as stressful. Items evaluate how unpredictable, uncontrollable, and overloaded respondents find their lives. Higher summed scores indicate greater perceived stress.

#### EMA of Eating Behavior

Participants received brief text-message prompts around mealtimes, 3 times per week (two weekdays, one weekend day), to complete a diet-reflection EMA. Each EMA asked participants to rate their hunger (very sated=1 to very hungry=7), mood (very depressed=1 to very elated=7), and anxiety (very calm=1 to very anxious=7) before and after eating, and to provide brief details about the meal (eg, location, meal type, and distractions while eating) [47,48]. Although these prompts captured real-time behavioral data, the primary purpose of EMA in this feasibility study was to assess participant engagement and responsiveness to frequent, real-time data collection procedures rather than to serve as the primary behavioral outcome measure.

#### DASH Diet Adherence

Dietary intake was collected using the MyFitnessPal mobile app (MyFitnessPal, Inc) to evaluate the feasibility of obtaining self-reported nutrient data in an eHealth intervention. Participants were trained to use the app’s barcode scanner, search functions, and recipe-entry tools and were asked to log their dietary intake for 3 days at baseline and after the intervention. From these logs, nutrient averages (eg, saturated fat, total fat, protein, cholesterol, fiber, sugar, calcium, potassium, and sodium) were converted into DASH adherence scores using the method of Mellen et al [49]. Scores ranged from 0 to 8, with higher values reflecting greater alignment with DASH dietary targets. In this feasibility study, these adherence scores primarily assessed the practicality of collecting complete, accurate diet records and calculating DASH-based nutrient metrics in an asynchronous eHealth program, rather than evaluating dietary effectiveness.

### Health Outcomes

#### Anthropometrics

Research staff, trained according to Centers for Disease Control and Prevention (CDC) body measurement guidelines, measured participants’ height using a stadiometer (Seca), weight using a digital scale (Seca), and waist and hip circumference with a flexible measuring tape. Equipment was calibrated according to the manufacturer’s instructions. Participants were asked to remove their shoes prior to height and weight measurement. Participants removed bulky clothing when necessary to permit accurate measurement as close to the skin as possible, and modesty gowns were offered when clothing interfered with accurate measurement. We took 3 measurements, averaged them, and calculated the waist-to-hip ratio as waist divided by hip circumference. BMI was calculated using the Adult BMI Calculator (Metric), developed by the CDC [50].

#### Blood Pressure

Blood pressure was recorded 3 times during morning visits using a Welch Allyn Spot Vital Signs 4400 automated cuff (Hillrom), and the average reading was calculated.

#### Blood Biospecimens

A trained phlebotomist collected 3 mL of fasting whole blood from participants via venipuncture of the left arm. Total cholesterol, triglycerides, low-density lipoproteins (LDLs), high-density lipoproteins (HDLs), and fasting glucose were analyzed using the Piccolo Xpress (Abaxis).

### Procedures

Potential participants completed an online screening questionnaire in Qualtrics (Qualtrics, LLC). Individuals who met the eligibility criteria received information about the study procedures and scheduled an early-morning, in-person baseline visit through an online scheduling system. Baseline and postintervention assessments were conducted in person at a university research site in the Southeastern United States. At the initial baseline visit, trained research staff reviewed the IRB-approved informed consent document, answered participant questions, and obtained written informed consent before initiating any study procedures. The consent process included discussion of study procedures, random assignment to one of two web-based nutrition education programs, study duration, potential risks and benefits, compensation, confidentiality, electronic data collection procedures, and participants’ right to withdraw at any time without penalty. Participants were informed that dietary data would be collected using MyFitnessPal and questionnaires would be administered through Qualtrics, both third-party platforms with their own privacy policies.

Participants then completed anthropometric measurements (height, weight, waist, and hip circumferences), fasting blood draws, blood pressure assessments, and self-report questionnaires. After the blood draw, participants received a snack. Baseline visits occurred approximately 1 week before the 6-week intervention began. Participants were trained to access the D2L Brightspace learning management system where the MYH program was housed and to use the MyFitnessPal mobile app to record dietary intake. Participants who did not already have a MyFitnessPal account received assistance creating a free account. They were instructed to complete 3-day food records (2 weekdays and 1 weekend day) at baseline and again during the final week of the intervention. Research staff also instructed participants on how to respond to EMA text messages. The intervention provided no additional behavioral counseling, individualized dietary guidance, or mindfulness coaching. Participants were instructed to continue their usual medical care, medications, and lifestyle practices throughout the study. Participation in other structured dietary or mindfulness programs and use of weight-loss medications were exclusion criteria. At the conclusion of the baseline visit, participants received a study packet with platform instructions, copies of their laboratory results, and a scheduled follow-up appointment. Baseline visits lasted approximately 45 minutes.

Apart from the pre- and postintervention visits, the intervention was fully automated and self-directed. Following baseline assessments and randomization, participants received a study-specific D2L Brightspace course linked to their university credentials and enrolled in their assigned course. Participants accessed the intervention remotely using their own internet-enabled computers or smartphones and completed the modules asynchronously at their convenience. Both groups completed 6 weekly online modules through the D2L platform, which was closed to participants outside the platform. The research team provided access by adding participants to the appropriate course (Diet only or MYH), with courses launching the following Monday after intake. They received email instructions and weekly reminders to log in, enhancing engagement. For research-related issues, participants could contact the research team via email or access a help page with the university IT contact information for technical support.

Participants in the Diet Only group received DASH diet education, including brief educational videos, written materials, quizzes, and goal-setting activities. Participants in the MYH group received the same DASH diet curriculum, plus additional guided mindful-eating exercises, audio practices, and reflective activities designed to increase awareness of hunger and satiety cues, eating pace, emotional triggers, and distracted eating. Participants were advised that completing the weekly intervention content, including audiovisual and written materials, interactive activities, and goal setting and reflection exercises, would take approximately 20‐30 minutes per week for the Diet Only group and 30‐45 minutes per week for the MYH group. These estimates reflected anticipated total module-completion time rather than audiovisual duration alone. Participants were instructed to complete each weekly module during the week it was released; however, previously released modules remained available throughout the intervention for review.

Participants completed the modules at their own pace and were encouraged to apply concepts in their daily lives. Both groups received standardized weekly reminder emails encouraging them to complete the current week’s module. Participants also received identical automated EMA prompts delivered by text message 3 times per week (2 weekdays and 1 weekend day). These prompts captured information on eating context, hunger, mood, and awareness during meals. Intervention engagement was monitored through module completion and EMA response rates. The intervention remained consistent throughout the 6-week period with no major technical disruptions reported.

At the end of the 6-week intervention, participants returned for a postintervention visit within 2 weeks of the end of the intervention period, during which all baseline measures were repeated. Research staff reviewed and recorded MyFitnessPal food records, and participants received copies of their laboratory values and a US $20 incentive upon completion. Multimedia Appendix 5 summarizes planned intervention exposure, including the number and timing of modules, reminders, EMA prompts, and participant support.

### Data Analysis

Data analysis was completed using SPSS (version 28; IBM Corp), with Little Missing Completely at Random (MCAR) tests performed in R version 4.6.1 (R Core Team, R Foundation for Statistical Computing) using the *naniar* package. Descriptive statistics summarized participant characteristics and feasibility outcomes. Continuous variables are presented as mean (SD) for normally distributed data or median (IQR) for nonnormally distributed data, whereas categorical variables are presented as frequencies and percentages. We assessed normality using the Shapiro-Wilk test and quantile–quantile (Q-Q) plots and evaluated homogeneity of variance using Levene test. Because only 2 linked pairs (1 couple and 1 coworker pair) were enrolled, statistical analyses did not adjust for clustering.

Because this was a pilot feasibility trial, analyses used complete cases for each outcome rather than an intent-to-treat approach. We evaluated missing data using Little MCAR test. Separate behavioral, dietary, and clinical outcomes tests were nonsignificant (behavioral outcomes, *χ*²_53_=62.58; *P*=.17; dietary outcomes, *χ*²_22_=18.34; *P*=.69; and clinical outcomes, *χ*²_77_=81.73; *P*=.34), indicating that missing-data patterns were consistent with MCAR. Therefore, we included participants with paired baseline and postintervention data for each respective outcome in the corresponding analyses, resulting in slight differences in sample sizes across outcomes. We did not perform multiple imputation because missingness was limited, data were consistent with MCAR, and the study was designed as an exploratory pilot feasibility trial. Feasibility outcomes were summarized using all participants relevant to each feasibility metric.

### Baseline Characteristics

The baseline characteristics were summarized for all enrolled randomized participants (n=37). Continuous variables were presented as mean (SD) when normally distributed or median (IQR) when nonnormally distributed. Categorical variables were presented as frequencies and percentages, with available responses as the denominator and missing responses reported where applicable. Exploratory between-group comparisons were conducted using independent-samples *t* tests or Mann-Whitney *U* tests for continuous variables. Categorical variables were compared using Pearson chi-square tests when expected cell count assumptions were met and Fisher exact tests when cell counts violated assumptions.

### Outcome Analysis

We calculated individual change scores (postintervention minus baseline) for all behavioral, dietary, and clinical outcomes. These change scores served as the metric for between-group comparisons. We used independent-samples *t* tests when assumptions were met and Welch *t* tests when homogeneity of variance was violated. Results are presented as mean differences with 95% CI and Hedges *g* standardized effect sizes. Although a priori hypotheses were directional, all statistical tests were 2-tailed with statistical significance defined as *P*<.05. Because outcome analyses were exploratory, no adjustment was made for multiple comparisons.

## Results

### Feasibility Outcomes

#### Overview of Feasibility Outcomes

We evaluated feasibility descriptively based on recruitment rates, retention, data collection completion, module participation, and EMA completion. Formal progression criteria thresholds were not specified a priori. Instead, we used outcomes to characterize the feasibility of the intervention and study procedures and identify areas needing refinement before future trials. Figure 1 displays completion information for each study measure.

**Figure 1.**
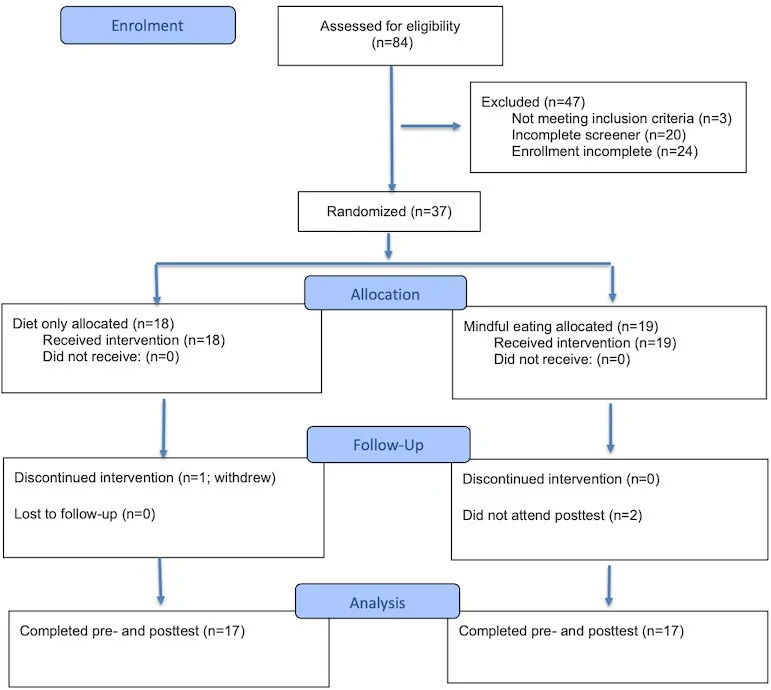
Participant flow from recruitment through analysis in the MYH 6-week randomized feasibility trial, January–May 2022. BP: blood pressure; MYH: Mind Your Heart.

#### Screening, Enrollment, and Retention

Recruitment occurred from January through March 2022. We sent recruitment emails to 42 managers across university units within a workplace of approximately 10,000 employees. Because managers could forward the invitation, we could not determine how many employees received or viewed the recruitment materials and therefore could not calculate a recruitment response rate.

Eighty-four people started the online eligibility screener. Of these, 47 individuals were not enrolled: 20 did not submit a completed screener or provide contact information, 3 did not meet the eligibility criteria, and 24 did not proceed with enrollment because they did not schedule or attend the enrollment appointment, canceled without rescheduling, or were unable to participate because of the study location. Ultimately, 37 participants provided informed consent, enrolled, and were randomized (Diet Only, n=18; MYH, n=19). Of the 37 randomized participants, 34 (91.9%) completed postintervention follow-up. One participant in the Diet Only group withdrew without providing a reason, and 2 participants in the MYH group were lost to follow-up after not attending their scheduled postintervention visits. Participants reported no adverse events, and research staff observed none during study procedures. Figure 1 presents the CONSORT (Consolidated Standards of Reporting Trials) (Checklist 1) flow diagram.

#### Data Collection Completion Rate

All 37 participants completed baseline questionnaires, anthropometric measurements, and blood pressure measurements. Blood lipid profiles were completed for 34 (91.9%) and metabolic profiles for 33 (89.2%), with 3 unable to provide blood despite 3 attempts.

After 1 participant withdrew, 36 participants were eligible for postintervention assessment. Of these, 34 (94.4%) completed the postintervention questionnaires, anthropometric measurements, and blood pressure measurements. Postintervention blood lipid profiles were available for 31 (86.1%) participants, and metabolic profiles were available for 30 (83.3%) participants. Paired baseline and postintervention questionnaire, anthropometric, and blood pressure data were available for 34 participants. Paired pre- and postintervention blood samples were available for 29 participants, with the analytic sample varying across individual laboratory outcomes because of outcome-specific missing data. Dietary records were available for 31 participants at baseline and 31 participants at postintervention. Because missing records occurred in different participants at the 2 time points, 30 participants had paired baseline and postintervention dietary records and were included in dietary outcome analyses.

#### Module Participation

Participants completed mean 85.9% (SD 13.1%) of the eHealth intervention content. They spent an average of 4 hours 56 minutes (SD 3 hours 45 minutes) engaging with the intervention over the 6-week study period. Participants in the Diet Only group spent an average of 3 hours 59 minutes (SD 2 hour 54 minutes) in D2L, whereas those in the MYH group spent 5 hours 52 minutes (SD 2 hours 49 minutes). They logged in an average of 6.22 days (SD 2.33). Overall, 31 of 36 participants (86.1%) completed at least 60% of their assigned curriculum. Additional engagement data are provided in Multimedia Appendix 5.

#### EMA Completion

Participants received EMA text messages 3 times per week (2 on weekdays, 1 on weekends). Among participants included in the EMA engagement analysis (N=36; 1 dropped out of the study), mean EMA completion was 77.5% (SD 22.6%), corresponding to a mean of 13.9 (SD 4.1) of 18 scheduled prompts per participant, with 32/36 (88.9%) completing at least half of the scheduled EMA prompts. Completion was highest on Tuesdays and Thursdays and lowest on Saturdays. Responses were mostly submitted around 10 AM (39/60, 72%); however, lunch was the most frequently reported meal (234/429, 54.5%).

#### Baseline Participant Characteristics

Participants reported a median age of 50 (IQR 39‐57), and most were female (34/37, 91.9%) and White (31/37, 83.8%). Tables 1 and 2 present baseline demographics and clinical characteristics by intervention for all participants who enrolled in the study and completed baseline measures. Significant between-group differences were observed for blood pressure diagnosis (*P*=.02), smoking history (*P=.*001), and alcohol drinks per week (*P*=.007). Those with hypertension or borderline hypertension, previous smokers, and more frequent alcohol drinkers were more likely to be in the MYH group. No other statistically significant baseline differences were observed.

**Table 1.** Baseline characteristics of participants in the 6-week MYH^a^ randomized feasibility trial, by study condition^b^.

Demographic characteristics	Total, N=37	Diet only, n=18	MYH, n=19	*P* values
Sex, n (%)				.60
Female	34 (91.9)	16 (88.9)	18 (94.7)	
Male	3 (8.1)	2 (11.1)	1 (5.3)	
Marital status, n (%)				.92
Married	28 (75.7)	13 (72.2)	15 (78.9)	
Widowed	1 (2.7)	1 (5.6)	—^c^	
Divorced	4 (10.8)	2 (11.1)	2 (10.5)	
Never married	4 (10.8)	2 (11.1)	2 (10.5)	
Race and ethnicity, n (%)				.69
White	31 (83.8)	14 (77.8)	17 (89.5)	
Black	2 (5.4)	1 (5.6)	1 (5.3)	
Asian	1 (2.7)	1 (5.6)	–—	
Other	3 (8.1)	2 (11.1)	1 (5.3)	
Hispanic, n (%)				>.99
Yes	4 (10.8)	2 (11.1)	2 (10.5)	
No	33 (89.2)	16 (88.9)	17 (89.5)	
Annual income (US $), n (%)				.08
$30,000 – -$39,999	8 (21.6)	7 (38.9)	1 (5.3)	
$40,000 – -$49,999	7 (18.9)	2 (11.1)	5 (26.3)	
$50,000 – -$59,999	10 (27)	4 (22.2)	6 (31.6)	
$60,000 – -$69,999	4 (10.8)	2 (11.1)	2 (10.5)	
$70,000 – -$79,999	—	—	—	
$80,000 – -$89,999	2 (5.4)	—	2 (10.5)	
$90,000 – -$99,999	2 (5.4)	2 (11.1)	—	
>$100,000	4 (10.8)	1 (5.6)	3 (15.8)	
Education, n (%)				.60
High school	1 (2.7)	1 (5.6)	––	
Some college	7 (18.9)	3 (16.7)	4 (21.1)	
2-years degree	7 (18.9)	5 (27.8)	2 (10.5)	
4-years degree	12 (32.4)	5 (27.8)	7 (36.8)	
Graduate or Professional	10 (27)	4 (22.2)	6 (31.6)	
Blood pressure diagnosis from a doctor, n (%)				.02
High blood pressure	10 (27)	3 (16.7)	7 (36.8)	
Borderline high blood pressure	4 (10.8)	––	4 (21.1)	
Normal blood pressure or not told	23 (62.2)	15 (83.3)	8 (42.1)	
Blood pressure medication, n (%)				.27
Yes	10 (27)	3 (16.7)	7 (36.8)	
No	27 (73)	15 (83.3)	12 (63.2)	
High cholesterol diagnosis from doctor, n (%)				.84
Yes	22 (59.5)	11 (61.1)	11 (57.9)	
No	15 (40.5)	7 (38.9)	8 (42.1)	
Cholesterol medication^d^, n (%)				.64
Yes	11 (29.7)	6 (33.3)	5 (26.3)	
No	26 (70.3)	12 (66.7)	14 (73.7)	
Diabetes diagnosis, n (%)				.09
Yes	4 (10.8)	4 (22.2)	—	
Borderline or prediabetic	5 (13.5)	3 (16.7)	2 (10.5)	
No	28 (75.7)	11 (61.1)	17 (89.5)	
Currently smoke, n (%)				—
Do not smoke	37 (100)	18 (100)	19 (100)	
Smoking history, n (%)				.001
Have ever smoked	9 (24.3)	––	9 (47.4)	
Never smoked	28 (75.7)	18 (100)	10 (52.6)	
Physical activity level, n (%)				.41
Sedentary	6 (18.2)	2 (13.3)	4 (22.2)	
Moderately active	9 (27.3)	6 (40)	3 (16.7)	
Active	18 (54.5)	7 (46.7)	11 (61.1)	
Missing physical activity level	4 (—)	3 (—)	1 (—)	

a MYH: Mind Your Heart.

b Participants were full-time employees recruited from a university workplace in the southeastern United States in 2022. Data are presented as n (%), mean (SD), or median (IQR) as appropriate. Percentages exclude missing observations. Between-group *P* values were calculated using Mann-Whitney *U* tests, Pearson *χ*2 tests, or exact tests, as appropriate.

c Not applicable.

d Total sample with blood cholesterol levels was 34 (Diet only n=17 and MYH n=17).

**Table 2.** Baseline anthropometric, clinical, and behavioral characteristics of participants by study condition.

Characteristic	Overall	Diet only	Mindful eating	*P* values^a^
Age (in years), median (IQR)	50 (39‐57)	45.5 (36.75‐56)	54 (46‐57)	.19
Alcohol consumption, median (IQR), drinks/week	1 (0‐6)	0 (0‐2.25)	4 (0‐12)	.007^b^
BMI, mean (SD), kg/m²	31.93 (7.25)	30.93 (6.01)	32.87 (8.31)	.42
Systolic blood pressure, mean (SD), mm Hg	123.67 (17.45)	121.93 (17.38)	125.33 (17.83)	.56
Diastolic blood pressure, mean (SD), mm Hg	77.82 (8.85)	77.52 (9.35)	78.10 (8.60)	.84
Total blood cholesterol^a^, mean (SD), mg/dL	189.96 (29.29)	188.06 (30.04)	191.85 (29.31)	.71

a P values represent between-group comparisons at baseline.

b *P*<.05.

### Behavioral Outcomes

#### Overview of Behavioral Outcomes

Because this was a feasibility study not designed to detect between-group differences, all statistical results should be viewed as exploratory and interpreted with caution. Statistical tests were only performed to identify potential signals for future trials and should not be taken as proof of effectiveness (Table 3 for behavioral outcomes).

**Table 3.** Changes in dietary behaviors, mindfulness, and stress from baseline to postintervention in the 6-week MYH^a^ randomized feasibility trial, by study condition^b^.

Behavior and group	N	Baseline, mean (SD)	Posttest, mean (SD)	Mean Δ (SD)	MYH − diet Δ (95% CI)	Hedges *g*^c^	*P* values^d^
Mindful awareness and attention					0.04 (−0.25 to 0.32)	0.08	.80
Diet only	17	4.44 (0.45)	4.29 (0.44)	−0.16 (0.34)			
MYH	17	4.38 (0.73)	4.26 (0.73)	−0.12 (0.47)			
Focused eating					−0.04 (−0.48 to 0.41)	−0.05	.87
Diet only	17	3.29 (0.82)	3.51 (0.86)	0.21 (0.79)			
MYH	17	3.15 (0.75)	3.33 (0.64)	0.18 (0.43)			
Hunger and satiety cues					0.26 (−0.08 to 0.60)	0.53	.13
Diet only	17	2.48 (0.70)	2.82 (0.66)	0.34 (0.46)			
MYH	16	2.27 (0.70)	2.88 (0.62)	0.60 (0.50)			
Eating with awareness					−0.16 (−0.68 to 0.37)	−0.20	.55
Diet only	17	3.96 (0.91)	4.16 (0.73)	0.20 (0.89)			
MYH	17	4.22 (1.01)	4.25 (0.86)	0.04 (0.58)			
Eating without distraction					0.23 (−0.26 to 0.72)	0.31	.36
Diet only	17	3.31 (0.65)	3.30 (0.67)	−0.01 (0.81)			
MYH	17	2.40 (0.88)	2.62 (0.85)	0.22 (0.58)			
Uncontrolled eating					−0.06 (−0.26 to 0.14)	−0.20	.56
Diet only	17	2.22 (0.54)	2.10 (0.58)	−0.12 (0.24)			
MYH	17	2.30 (0.53)	2.12 (0.53)	−0.18 (0.33)			
Cognitive restraint					0.39 (0.06 to 0.72)	0.81	.02
Diet only	17	2.79 (0.42)	2.68 (0.47)	−0.12 (0.36)			
MYH	**16**	2.57 (0.43)	2.84 (0.41)	0.27 (0.56)			
Emotional eating					−0.15 (−0.45 to 0.15)	−0.34	.32
Diet only	17	2.50 (0.86)	2.40 (0.89)	−0.10 (0.40)			
MYH	17	2.32 (0.68)	2.08 (0.71)	−0.25 (0.46)			
Perceived stress scale					1.06 (−1.92 to 4.04)	0.25	.47
Diet only	17	16.24 (7.00)	13.94 (7.49)	−2.29 (5.27)			
MYH	17	18.06 (8.23)	16.82 (8.91)	−1.24 (2.77)			
Autonomous regulation					3.82 (−2.50 to 10.15)	0.41	.23
Diet only	17	60.41 (15.69)	64.53 (10.44)	4.12 (10.58)			
MYH	17	62.88 (10.39)	70.82 (6.93)	7.94 (7.20)			
Controlled regulation					3.59 (0.01 to 7.16)	0.68	.049
Diet only	17	31.65 (12.32)	29.47 (9.50)	−2.18 (5.47)			
MYH	17	30.71 (11.12)	32.12 (12.23)	1.41 (4.74)			
Total regulation score					0.24 (−6.53 to 7.00)	0.02	.94
Diet only	17	28.76 (16.53)	35.06 (12.13)	6.29 (10.57)			
MYH	17	32.18 (17.62)	38.71 (15.54)	6.53 (8.72)			

a MYH: Mind Your Heart.

b Participants were full-time employees recruited from a university workplace in the southeastern United States in 2022. Values are mean (SD). Change (Δ) was calculated as postintervention minus baseline. Analyses included participants with complete baseline and postintervention data for the respective outcome; therefore, sample sizes vary by outcome. Between-group differences represent mean change in MYH minus mean change in diet only and are presented with 95% CIs. We used Welch *t* test when homogeneity of variance was violated.

c Hedges *g* represents the standardized between-group difference in change.

d All reported *P* values are 2-sided.

#### Mindful Eating Behaviors

No statistically significant between-group differences were observed for focused eating, hunger and satiety cues, eating with awareness, or eating without distraction (all *P*>.05). The largest between-group effect was observed for hunger and satiety *c*ues (Hedges *g*=0.53), although the difference did not reach statistical significance (*P*=.13).

#### Three-Factor Eating Questionnaire

Participants in the MYH group showed significantly greater improvement in cognitive restraint than those in the Diet Only group (mean difference in change 0.39, 95% CI 0.06-0.72; Hedges *g*=0.81; *P*=.02). No significant between-group differences were observed for uncontrolled eating or emotional eating*.*

#### Motivation for Regulated Eating

Participants in the MYH group showed significantly greater improvement in controlled regulation than the Diet Only group (mean difference in change 3.59, 95% CI 0.01-7.16; Hedges *g*=0.68; *P*=.05). No significant between-group differences were observed for autonomous regulation or total regulation scores.

#### Perceived Stress

No statistically significant between-group difference was observed in change in perceived stress (mean difference in change 1.06, 95% CI −1.92 to 4.04; Hedges *g*=0.25; *P*=.47).

#### EMA Eating Behavior

Among the 518 meal-feeling responses, participants most frequently reported feeling relaxed during meals (356/518, 68.7%). The MYH group more frequently reported feeling distracted (33/253, 13.0%) and rushed (40/253, 15.8%) and less frequently reported feeling relaxed (166/253, 65.6%) than the Diet Only group, whereas reports of feeling stressed were uncommon in both groups. Among the 499 responses with meal location data, most responses were completed at home (215/499, 43.1%) or at work or at a desk (176/499, 35.3%). Participants in the Diet Only group more frequently reported eating in restaurants or dining halls than participants in the MYH group (28/256, 10.9% vs 16/243, 6.6%). Additional descriptive EMA engagement and meal characteristics are presented in Multimedia Appendix 6.

### DASH Diet Changes

Table 4 presents dietary outcomes. At baseline, the MYH and Diet Only groups had similar dietary intake across most nutrients and DASH diet scores, although the MYH group reported higher calcium intake than the Diet Only group. No statistically significant between-group differences were observed in changes in caloric intake, macronutrients, micronutrients, or overall DASH diet adherence over the 6-week intervention (all *P*>.05).

**Table 4.** Changes in caloric and nutrient intake from baseline to postintervention in the 6-week MYH^a^ randomized feasibility trial, by study condition.

Outcome and group	Baseline, mean (SD)	Posttest, mean (SD)	Mean Δ^b^ (SD)	MYH − diet Δ (95% CI)	Hedges *g^c^*	*P* values^d^
Calories (kcal per day)				118.21 (−194.77 to 431.19)	0.28	.45
Diet only	1573.24 (661.75)	1451.53 (523.29)	−121.71 (472.78)			
MYH	1703.52 (572.03)	1700.02 (686.85)	−3.50 (355.89)			
Protein (g per day)				−10.06 (−27.27 to 7.16)	−0.43	.24
Diet only	64.36 (24.17)	65.93 (22.80)	1.58 (25.77)			
MYH	76.22 (18.69)	67.74 (26.77)	−8.48 (19.87)			
Fiber (g per day)				3.39 (−1.70 to 8.47)	0.49	.18
Diet only	17.73 (10.45)	16.51 (10.28)	−1.22 (7.61)			
MYH	18.48 (10.69)	20.64 (8.50)	2.17 (5.88)			
Sugar (g per day)				−9.29 (−24.16 to 5.58)	−0.46	.21
Diet only	47.76 (33.33)	50.84 (38.66)	3.09 (16.94)			
MYH	59.12 (37.15)	52.92 (23.52)	−6.20 (22.44)			
Total fat (g per day)				10.31 (−13.87 to 34.49)	0.31	.39
Diet only	66.02 (42.56)	61.24 (25.23)	−4.78 (31.80)			
MYH	75.47 (26.50)	81.00 (48.63)	5.53 (32.84)			
Saturated fat (g per day)				−3.16 (−8.73 to 2.42)	−0.41	.26
Diet only	19.36 (13.60)	20.93 (10.86)	1.58 (8.14)			
MYH	22.68 (10.74)	21.10 (10.34)	−1.58 (6.69)			
Cholesterol (mg per day)				−65.89 (−158.58 to 26.80)	−0.52	.16
Diet only	154.44 (134.28)	197.69 (116.61)	43.24 (117.53)			
MYH	223.84 (118.90)	201.20 (143.10)	−22.64 (130.00)			
Sodium (mg per day)				−421.49 (−956.89, to 113.91)	−0.57	.12
Diet only	1975.13 (824.12)	2125.91 (776.95)	150.78 (771.01)			
MYH	2374.02 (1136.65)	2103.31 (724.75)	−270.71 (655.96)			
Potassium (mg per day)				54.22 (−343.34 to 451.79)	0.1	.78
Diet only	1202.20 (854.77)	979.76 (679.74)	−222.44 (591.67)			
MYH	1364.86 (787.83)	1196.63 (665.02)	−168.22 (463.63)			
Calcium (mg per day)				−46.51 (−97.53 to 4.50)	−0.66	.07
Diet only	52.33 (34.16)	66.13 (51.71)	13.80 (55.16)			
MYH	123.79 (101.87)	91.08 (71.55)	−32.71 (79.13)			
DASH^e^ score				0.57 (−0.38 to 1.51)	0.44	.23
Diet only	2.73 (1.27)	2.87 (1.23)	0.13 (1.04)			
MYH	2.57 (1.22)	3.27 (0.90)	0.70 (1.45)			

a MYH: Mind Your Heart.

b Δ=post-intervention − baseline. Between-group comparisons were performed using independent-samples *t* tests of change scores. We used Welch *t* test when the homogeneity-of-variance assumption was violated.

c Effect sizes are reported as Hedges *g*.

d All *P* values are 2-sided.

e DASH: Dietary Approaches to Stop Hypertension.

### Health Outcomes

Anthropometric and blood pressure outcomes were available for 17 participants per group. Laboratory outcome sample sizes ranged from 14 to 15 participants per group because of missing follow-up laboratory assessments. No statistically significant between-group differences were observed for changes in anthropometric measures, systolic or diastolic blood pressure, or cardiometabolic biomarkers, including total cholesterol, HDL cholesterol, LDL cholesterol, triglycerides, and fasting glucose (Table 5).

**Table 5.** Changes in anthropometric and cardiometabolic outcomes from baseline to postintervention in the 6-week MYH^a^ randomized feasibility trial, by study condition^b^.

Behavior and group	N	Baseline, mean (SD)	Posttest, mean (SD)	Mean Δ^b^ (SD)	MYH − diet Δ (95% CI)	Hedges *g^c^*	*P* values^d^
Weight (kg)					−1.34 (−3.20 to 0.53)	−0.49	.15
Diet only	17	82.30 (18.76)	81.31 (19.06)	−0.99 (1.32)			
MYH^a^	17	90.32 (26.55)	87.99 (26.04)	−2.33 (3.54)			
Waist circumference (cm)					−0.47 (−3.07 to 2.12)	−0.12	.71
Diet only	17	94.64 (14.43)	94.29 (16.02)	−0.35 (3.50)			
MYH	17	95.36 (20.69)	94.53 (20.09)	−0.82 (3.92)			
Hip circumference (cm)					0.00 (−2.49 to 2.50)	0.00	>.99
Diet only	17	111.00 (13.05)	110.14 (13.85)	−0.86 (2.40)			
MYH	17	117.35 (16.78)	116.49 (17.21)	−0.86 (4.44)			
BMI (kg/m²)					−0.18 (−0.60 to 0.24)	−0.30	.38
Diet only	17	30.31 (5.57)	29.91 (5.72)	−0.41 (0.49)			
MYH	17	32.29 (8.61)	31.70 (8.33)	−0.59 (0.69)			
Waist-to-hip ratio					−0.010 (−0.031 to 0.011)	−0.32	.35
Diet only	17	0.852 (0.072)	0.853 (0.067)	0.001 (0.025)			
MYH	17	0.815 (0.094)	0.805 (0.089)	−0.009 (0.035)			
Systolic blood pressure (mm Hg)					5.50 (−2.39 to 13.40)	0.48	.17
Diet only	17	121.86 (17.91)	113.23 (17.77)	−8.64 (8.90)			
MYH	17	123.31 (16.93)	120.18 (16.48)	−3.13 (13.27)			
Diastolic blood pressure (mm Hg)					3.03 (−1.19 to 7.24)	0.49	.15
Diet only	17	77.43 (9.63)	73.92 (9.61)	−3.51 (5.83)			
MYH	17	77.02 (8.28)	76.53 (8.02)	−0.49 (6.22)			
Total cholesterol (mg per dL)					1.97 (−8.30 to 12.24)	0.14	.70
Diet only	15	189.00 (31.99)	186.07 (30.20)	−2.93 (9.31)			
MYH	15	189.97 (30.48)	189.00 (25.55)	−0.97 (16.77)			
HDL^e^ cholesterol (mg/dL)					2.26 (−1.03 to 5.55)	0.51	.17
Diet only	15	67.13 (15.74)	63.73 (15.41)	−3.40 (4.69)			
MYH	14	61.71 (10.48)	60.57 (11.35)	−1.14 (3.88)			
Triglycerides (mg/dL)					3.37 (−27.28 to 34.01)	0.08	.82
Diet only	15	116.67 (50.18)	114.53 (53.09)	−2.13 (51.75)			
MYH	15	109.17 (58.38)	110.40 (56.25)	1.23 (23.42)			
Glucose (mg/dL)					4.13 (−7.37 to 15.64)	0.26	.47
Diet only	15	110.87 (34.26)	108.20 (24.25)	−2.67 (20.89)			
MYH	15	104.20 (12.18)	105.67 (12.56)	1.47 (6.07)			
LDL^f^ cholesterol (mg/dL)					−3.94 (−12.16 to 4.28)	−0.36	.33
Diet only	15	98.60 (25.92)	99.40 (27.50)	0.80 (9.86)			
MYH	14	101.93 (24.81)	98.79 (25.40)	−3.14 (11.69)			

a MYH: Mind Your Heart.

b Values are presented as mean (SD). Change (Δ) was calculated as postintervention minus baseline. Analyses included participants with complete baseline and postintervention data for the respective outcome; therefore, sample sizes varied by outcome. Between-group differences represent mean change in MYH minus mean change in diet only and are presented with 95% CIs.

c Hedges *g* represents the standardized between-group difference in change. Between-group comparisons were conducted using independent-samples *t* tests; Welch *t* test was used when the homogeneity-of-variance assumption was violated.

d All *P* values are 2-sided.

e HDL: high-density lipoproteins.

f LDL: low-density lipoproteins.

## Discussion

### Principal Findings

This feasibility study showed that a fully asynchronous digital mindful eating program integrated with DASH diet education could be implemented successfully, with high participant engagement and retention. Participants completed an average of 85.9% (SD 21.4) of the assigned curriculum, and EMA completion reached 77.5%, supporting the feasibility of repeated real-time dietary assessment in this population. Exploratory between-group analyses suggested that participants assigned to MYH experienced greater improvements in cognitive restraint and controlled regulation than those assigned to the Diet Only group. No statistically significant between-group differences were observed for DASH adherence, nutrient intake, anthropometric measures, blood pressure, or cardiometabolic biomarkers after the 6-week intervention.

### Comparison With Prior Work

#### Feasibility and Intervention Engagement

The primary objective of the study was to assess the feasibility of delivering mindful eating training combined with DASH diet education via eHealth. We hypothesized that participants would complete most study measures and engage with most of the content, despite the absence of “high-touch” in-person interactions and the additional time required for mindful eating exercises in the mindful eating group. Participant retention, curriculum completion, and EMA compliance suggest that the intervention and study procedures were feasible among employed participants. Participants completed an average of 85.9% of their assigned curriculum. Our module percent completion rates of 80%‐94% surpass typical eHealth adherence rates (30%‐60%) reported in a systematic review [51], likely due to ongoing engagement through EMA three times weekly and reminder emails.

The EMA completion rate of 77.5% further supports the feasibility of collecting repeated, real-time data on eating behavior in this population, while identifying opportunities to optimize prompt timing and engagement in future trials. Consistent responses on Tuesdays, Thursdays, and mornings suggest optimal engagement times and will guide future efforts. Although our rates suggest high engagement, other studies report higher EMA compliance with incentives [52]. Although EMA completion was not directly linked to mindfulness, the EMA data highlighted eating contexts and emotional states. Previous research suggests that EMA can trigger mindfulness and influence behavior [53]. These results highlight EMA’s potential to enhance dietary awareness and to identify contextual adherence barriers.

These findings inform the refinement of future larger trials by emphasizing retention rates, engagement with the intervention, participant responsiveness to EMA, completion of multimodal assessments, and highlighting aspects of the recruitment and data-collection protocols that might require adjustments.

#### Behavioral and Dietary Outcomes

Although this pilot study was not powered to detect efficacy, participants assigned to the MYH group demonstrated significant changes in cognitive restraint and controlled regulation compared to the Diet Only group over 6-weeks. These findings are consistent with previous research suggesting that mindful eating may improve eating-related self-regulation and motivation [54,55]. Although between-group differences were not statistically significant for perceived stress and other behavioral outcomes, the observed patterns may provide useful estimates for fully powered randomized trials. However, given the small sample size, interpret these observations with caution.

EMA responses suggested that participants in the MYH group reported distraction during meals more frequently than the Diet Only group. Because these data were summarized descriptively and repeated observations were not modeled statistically, interpret these findings cautiously. One possible explanation is that mindfulness training increased participants’ awareness of distractions during eating rather than the frequency of distraction itself. EMA has been used in health interventions like smoking and alcohol cessation, helping participants identify negative triggers [56]. Mindfulness training may increase awareness of automatic distractions outside conscious awareness [57], leading to more reports of distraction even as distracted eating decreases over time. However, future studies must confirm this hypothesis.

No significant differences were observed between groups in DASH adherence or nutrient intake after the 6-week intervention. While some outcomes favored the MYH group, these exploratory results should be viewed cautiously due to the small sample size and feasibility focus. These findings align with previous mindful eating studies [58-60] showing mixed effects on diet quality, often comparable to standard dietary education. Larger, longer studies are needed to confirm if mindful eating offers meaningful benefits for DASH adherence and dietary quality.

#### Clinical Changes in Physical Health

This study aimed to explore initial differences between groups in anthropometric and cardiometabolic results after a six-week intervention. The study found no significant differences in weight, BMI, waist and hip circumference, blood pressure, or biomarkers. Both groups showed minor improvements, but the study lacked the power to assess if these were clinically significant. Baseline differences, especially in cardiometabolic risk factors, complicate interpretation; the MYH group had higher hypertension prevalence and antihypertensive medication use, impacting blood pressure responses. Small baseline variances in anthropometrics reflect typical pilot study variability. Larger, longer-term trials with balanced groups are needed to determine if mindful eating combined with digital DASH education improves cardiometabolic health.

### Strengths and Limitations

This study has several important limitations. First, registration on ClinicalTrials.gov was done retrospectively, after participant enrollment began. Nonetheless, the study’s objectives, intervention procedures, eligibility criteria, and outcome measures were all predefined in an IRB-approved protocol before recruitment started. The retrospective registration primarily enhanced transparency and public access. As a pilot feasibility study, it was not designed to establish efficacy, so all outcome results are exploratory. Additionally, the small sample size hindered the detection of differences between groups and limited our ability to control for baseline variations in hypertension, medication use, and other cardiometabolic risk factors.

Second, 4 participants comprised 2 linked pairs (1 couple and 1 pair of coworkers). To minimize contamination, we randomized the first participant in each pair and assigned the second to the same study condition. Because only 2 linked pairs were enrolled, statistical analyses did not account for within-pair clustering, although any resulting bias is expected to be minimal.

Third, dietary intake was self-reported using MyFitnessPal. Because the platform does not provide all nutrients required for the original DASH Accordance Score (eg, magnesium), we used a modified DASH score, and self-reported dietary data remain subject to recall and reporting bias. The 6-week intervention, absence of long-term follow-up, and predominantly White, female, and middle- to upper-income university employees also limit the generalizability of these findings. Both groups also completed repeated EMA prompts and dietary self-monitoring, which may themselves have increased awareness of eating behavior or influenced dietary choices. The Diet Only condition represented an active comparison condition that included DASH education and repeated self-monitoring procedures rather than DASH education alone. The potential behavioral reactivity associated with EMA and dietary recording should therefore be considered when interpreting between-group differences.

Finally, analyses used available paired observations for each outcome rather than an intention-to-treat approach. Although missing data were consistent with missing completely at random, excluding participants with missing outcome data may still have introduced bias. Future adequately powered randomized controlled trials should prospectively register study protocols, use intention-to-treat analyses with appropriate missing-data methods, recruit more diverse populations, and include longer follow-up periods to evaluate sustained intervention effects.

Despite these limitations, this study has several strengths. To our knowledge, it is among the first feasibility trials to integrate mindful eating with DASH diet education in a fully asynchronous digital intervention. Participant retention, curriculum completion, and EMA adherence demonstrated the feasibility of both the intervention and study procedures. The study also incorporated behavioral, dietary, anthropometric, and biomarker assessments to provide a comprehensive evaluation of feasibility and preliminary intervention effects. Finally, stratified randomization based on trait mindfulness strengthened the study design and may inform future definitive trials.

### Conclusions

This feasibility trial demonstrated that a fully asynchronous digital mindful eating plus DASH diet education intervention and associated research procedures were feasible to implement and achieved high participant engagement and retention among enrolled participants. Exploratory between-group findings suggest possible differences in eating behaviors between diet-only education and the mindful eating condition, but these findings should be interpreted with caution due to the small sample size and feasibility design. These preliminary findings support further evaluation of mindful eating as a component of digital DASH diet interventions in adequately powered randomized controlled trials with longer follow-up.

## Supplementary material

10.2196/106388 Multimedia Appendix 1 Comparison of the original study protocol, submitted paper, and revised analytic approach.

10.2196/106388 Multimedia Appendix 2 Representative intervention materials.

10.2196/106388 Multimedia Appendix 3 Six-Week Mind Your Heart (MYH) Dietary Approaches to Stop Hypertension (DASH) nutrition curriculum provided to both study conditions.

10.2196/106388 Multimedia Appendix 4 Six-week Mind Your Heart (MYH) additional mindful-eating curriculum provided to the MYH condition.

10.2196/106388 Multimedia Appendix 5 Intervention delivery, planned exposure, and actual exposure.

10.2196/106388 Multimedia Appendix 6 Ecological Momentary Assessment (EMA) engagement and meal characteristics.

10.2196/106388 Checklist 1 CONSORT checklist.

## Abbreviations

CDC: Centers for Disease Control and Prevention
CONSORT: Consolidated Standards of Reporting Trials
CVD: cardiovascular disease
D2L: Desire2Learn
DASH: Dietary Approaches to Stop Hypertension
E-RYT 500: experienced registered yoga teacher at the 500 hour level
EMA: ecological momentary assessment
HDL: High-density lipoprotein
IRB: Institutional Research Board
LDL: Low-density lipoprotein
MAAS: Mindful Attention Awareness Scale
MB-BP: Mindfulness-Based Blood Pressure
MCAR: Missing Completely at Random
MEBS: Mindful Eating Behaviors Scale
MYH: Mind Your Heart
PSS-10: Perceived Stress Scale 10-item
Q-Q: quantile–quantile
REBS: Regulated Eating Behaviors Scale
SMART: specific, measurable, achievable, realistic, time frame
TEFQ-R21: Three-Factor Eating Questionnaire-Revised 21 item
