# Supplementary material for: Mind Your Heart—A Web-Based Dietary Approaches to Stop Hypertension Diet and Mindful Eating Intervention for Working Adults at Elevated Cardiovascular Risk: Pilot Randomized Controlled Trial
Source: JMIR Form Res. 2026 Oct 28;10:e106388. doi: 10.2196/106388 (PMC13716348; doi:10.2196/106388)
Supplement: Supplementary file 2 [file formative-v10-e106388-s001.docx]

**Multimedia Appendix 6:** Comparison of the Original Study Protocol, Submitted Manuscript, and Revised Analytic Approach

| Study element | Original IRB-approved protocol | Submitted manuscript | Revised manuscript | Change/rationale |
| --- | --- | --- | --- | --- |
| Study objectives | Assess the feasibility and preliminary effects of a 6-week eHealth DASH diet intervention with mindful eating. | Same. | Same. | No substantive changes. Objectives clarified for consistency with feasibility design. |
| Target sample size | 40 participants (pilot feasibility study). | Reported enrolled sample (N = 37). | Same. | No change. Three participants withdrew or were lost before follow-up. |
| Eligibility criteria | full-time employment, English fluency, and internet access.  Exclusions were pregnancy, specialized diets for chronic illness, weight-loss medications, or enrollment in other dietary or mindfulness programs. | Same. | Same. | No changes |
| Primary outcomes | Feasibility (recruitment, retention, engagement, adherence) and exploratory changes in behavioral, dietary, and clinical outcomes. | Behavioral, dietary, and clinical outcomes emphasized. | Feasibility outcomes emphasized; exploratory between-group comparisons retained. | Revised to better reflect the pilot feasibility objectives. |
| Secondary outcomes | Anthropometrics, blood pressure, laboratory biomarkers, EMA engagement, behavioral questionnaires. | Same. | Same. | No substantive changes. |
| Analysis population | Participants completing study procedures with available outcome data. | Restricted primarily to participants with complete paired diet records (n = 30). | Available-case analyses performed separately for each outcome using participants with complete data for that outcome. | Revised following peer review to avoid excluding participants because of missing data on unrelated measures. |
| Statistical procedures | Descriptive feasibility analyses and exploratory between-group comparisons. | Mixed use of complete-case analyses and within-group testing. | Available-case analyses, emphasis on between-group comparisons, effect sizes with 95% CIs, Little's MCAR assessment, no multiple imputation. | Revised to improve transparency and align analyses with reviewer recommendations. |
| Missing data handling | Not explicitly specified. | Listwise deletion across primary analyses. | Missingness evaluated using Little's MCAR test; available-case analyses conducted for each outcome; multiple imputation not performed because missingness was limited and consistent with MCAR in this pilot study. | Added in response to reviewer request for transparent reporting of missing data. |
| Trial registration | Not prospectively registered. | Retrospectively registered during peer review. | ClinicalTrials.gov registration reported throughout manuscript. | Trial registered retrospectively on June 26, 2026, after completion of data collection. |
