# Supplementary material for: Mind Your Heart—A Web-Based Dietary Approaches to Stop Hypertension Diet and Mindful Eating Intervention for Working Adults at Elevated Cardiovascular Risk: Pilot Randomized Controlled Trial
Source: JMIR Form Res. 2026 Oct 28;10:e106388. doi: 10.2196/106388 (PMC13716348; doi:10.2196/106388)
Supplement: Supplementary file 3 [file formative-v10-e106388-s002.docx]

**Multimedia Appendix 1:** Representative Intervention Materials

**Figure 1.1** Mind Your Heart Module 1 Example Layout

**Figure 1.2** Mind Your Heart module content page with video content screenshot

**Figure 1.3** Mind Your Heart interactive video quiz example screenshot

**Figure 1.4** Mind Your Heart guided activity page example with video screenshot

**Figure 1.5** Mind Your Heart goal tracking exercise example screenshot
