# Supplementary material for: Mind Your Heart—A Web-Based Dietary Approaches to Stop Hypertension Diet and Mindful Eating Intervention for Working Adults at Elevated Cardiovascular Risk: Pilot Randomized Controlled Trial
Source: JMIR Form Res. 2026 Oct 28;10:e106388. doi: 10.2196/106388 (PMC13716348; doi:10.2196/106388)
Supplement: Supplementary file 4 [file formative-v10-e106388-s003.docx]

**Multimedia Appendix 2**: Six-Week Mind Your Heart DASH Nutrition Curriculum Provided to Both Study Conditions

| **Week** | **Module** | **Submodule name used in D2L template** | **Objective used in D2L template** | **Activities / reflective tasks** | **Video Duration (mm:ss)** |
| --- | --- | --- | --- | --- | --- |
| **1** | **Healthy Diet and Disease Prevention** | 1.a Overview of the DASH Diet | Identify the components of the DASH diet. | Interactive knowledge check; | 4:39 |
|  |  | 1.b How Diet Prevents Disease | Relate diet quality with heart health outcomes. | Interactive knowledge check | 6:59 |
|  |  | 1.c SMART Goals | Create personalized SMART goals for monitoring progress | Write weekly nutrition goal setting exercise | 5:54 |
| **2** | **Balanced Diet** | 2.a What Makes a Diet Nutritious? | Differentiate between macro- and micronutrients. | Interactive knowledge check; weekly nutrition goal reflection and goal setting | 6:33 |
|  |  | 2.b Food Groups | Categorize foods into food groups. | Interactive knowledge check | 5:15 |
|  |  | 2.c Portion Control | Demonstrate portion control using evidence-based tools such as MyPlate. | Interactive knowledge check and applied portion-control activity | 3:38 |
|  |  | 2.d Water and Fluid Intake | Select a low sugar option for hydration; identify the appropriate number ounces of water per body weight. | Interactive knowledge check | 7:09 |
|  |  | 3.e Alcohol and Health | Identify the appropriate servings of alcohol for heart health | Interactive knowledge check | 5:44 |
| **3** | **Planning Meals** | 3.a What’s in a Nutrition Label? | Locate the essential nutrients and macronutrients on a food label. | Interactive knowledge check; nutrition-label application; weekly nutrition goal reflection and goal setting | 4:11 |
|  |  | 3.b Cooking Tips and Tricks | Identify cooking methods to better adhere to the DASH diet. | Interactive knowledge check; application of DASH-compatible cooking methods/substitutions | 4:07 |
|  |  | 3.c Recipes and Menu Planning | Construct a meal plan schedule for different recipes and meals for 7 days. | Meal-planning demonstration/activity | 4:44 |
|  |  | 3.d Snacking and Desserts | Select low sugar and/or sodium alternatives for snacks and desserts to satisfy cravings. | Interactive knowledge check; food substitution activity | 3:51 |
| **4** | **Dining Away from Home** | 4.a Eating at Work | Plan methods to maintain the DASH diet while at work (eg, bring meals from home or find food that adheres to the diet plan). | Applied planning activity; weekly nutrition goal reflection and goal setting | 3:32 |
|  |  | 4.b Dining at Restaurants | Plan methods for DASH diet adherence using nutrition labels and caloric counts on menus. | Menu-selection/application activity | 3:54 |
|  |  | 4.c Work Functions and Parties | Identify methods of portion control for discretionary foods, such as the MyPlate method and other tools. | Applied food-selection/portion-control activity | 3:21 |
| **5** | **Healthy Lifestyle Changes** | 5.a Healthy Eating for Life: Changes for a Sustainable Lifestyle | Create goals for sustaining a DASH diet eating plan throughout the lifespan. | Interactive knowledge check; goal reflection and weekly nutrition goal setting | 5:30 |
|  |  | 5.b The Truth About Fad Diets | Identify typical issues in fad diets. | Interactive knowledge check | 9:39 |
|  |  | 5.c Other Lifestyle Changes | Identify additional methods to sustain a healthy lifestyle. | Interactive knowledge check; application to physical activity and other lifestyle behaviors | 10:17 |
| **6** | **Help and Social Support** | 6.a Consult Doctor, Nutritionist, Health Coach, or Personal Trainer | Locate a doctor, nutritionist, or other health professional for help and support. | Resource-identification activity; final nutrition goal reflection and future goal setting | 5:02 |
|  |  | 6.b Social Support | Identify a social support group to live a healthy lifestyle. | Social-support identification/reflection | 4:04 |
|  |  | 6.c References and Resources | Identify reputable references and resources to continue the DASH diet lifestyle. | Evaluation/identification of reputable health resources | 3:46 |

**Note.** D2L = Desire2Learn; DASH = Dietary Approaches to Stop Hypertension. Video duration reflects the duration of recorded instructional content and does not include time spent reading module materials, completing embedded knowledge checks, behavioral activities, goal-setting exercises, or other self-directed activities. Because the intervention was asynchronous, total time required to complete each module varied by participant. Modules were released weekly and previously released content remained accessible through the end of the six-week intervention.
