# Supplementary material for: Mind Your Heart—A Web-Based Dietary Approaches to Stop Hypertension Diet and Mindful Eating Intervention for Working Adults at Elevated Cardiovascular Risk: Pilot Randomized Controlled Trial
Source: JMIR Form Res. 2026 Oct 28;10:e106388. doi: 10.2196/106388 (PMC13716348; doi:10.2196/106388)
Supplement: Supplementary file 5 [file formative-v10-e106388-s004.docx]

**Multimedia Appendix 3**: Six-Week Mind Your Heart Additional Mindful-Eating Curriculum Provided to the MYH Condition

| **Week** | **Module** | **Submodule name used in D2L template** | **Objective used in D2L template** | **Exercise / reflective task** | **Duration** |
| --- | --- | --- | --- | --- | --- |
| **1** | **Introduction to Mindful Eating** | ME 1.a What is Mindfulness and How Does it Work? | Define mindfulness and how it is related to behaviors. | Reflection on definition/application of mindfulness | 2:47 |
|  |  | ME 1.b What is Mindful Eating? | Differentiate between mindful and mindless eating. | Reflect on mindful vs mindless eating and strategies for increasing awareness while eating | 6:33 |
|  |  | ME 1.c Clarifying your values | Define values and goals related to health. | Identify and clarify personal values related to food and health | 4:00 |
|  |  | ME 1.d Activity: Formal Guided Breathing Awareness | Participate in formal self-awareness practice. | Guided breathing practice; establish a practice goal and reflect on use of the practice | 5:16 |
| **2** | **Internal vs External Hunger Cues** | ME 2.a Mindfulness and the Everyday Adult | Identify how mindfulness applies to everyday tasks | Reflection on mindfulness in everyday life | 4:40 |
|  |  | ME 2.b Solutions to Types of Hunger | Provide examples of solutions for each one of the types of hunger. | Identify source(s) of hunger and appropriate responses to eye, nose, mouth, stomach, cellular, mind, and heart hunger | 7:57 |
|  |  | ME 2.c Activity: Guided Eating Awareness Exercise | Participate in a guided eating awareness exercise. | Assess hunger before and after guided eating-awareness practice; weekly mindful-eating goal reflection and goal setting | 8:43 |
| **3** | **Habits and Patterns with Food** | ME 3.a Conditioned Behaviors | Increase confidence to recognize emotional responses to typical food behaviors. | Identify conditioned/emotional responses associated with eating | 6:49 |
|  |  | ME 3.b Guided Emotional Awareness Activity | Increase awareness of conditioned eating responses through a guided mindful practice. | Guided emotional-eating awareness practice; reflection on stress, emotions, cravings, and eating; weekly goal setting | 7:15 |
| **4** | **Food Environ-ments** | ME 4.a. Becoming Mindful of Environmental Triggers | Identify environmental factors that affect food behaviors. | Identify personal environmental eating triggers | 6:56 |
|  |  | ME 4b Activity: Paying Attention to the Food Setting | Participate in a mindful environment awareness exercise. | Guided awareness of eating setting/context; weekly mindful-eating goal reflection and goal setting | 4:29 |
| **5** | **Satiety Cues** | ME 5.a Being Aware of Bodily Sensations | Describe how awareness impacts the sensations of the body. | Reflection on bodily sensations associated with eating, hunger, and fullness | 5:44 |
|  |  | ME 5.b Activity: Body Scan | Participate in a mindful body scan. | Guided body scan; reflection on bodily sensations; weekly mindful-eating goal reflection and goal setting | 7:01 |
| **6** | **Revisiting Values** | ME 6a. Re-establishing Values Surrounding Food | Compare pre-intervention values to post-intervention values as it relates to food behaviors. | Revisit initial food/health values and goals; reflect on changes and establish future goals | 4:26 |
|  |  | ME 6b. Activity: Gratitude for Food and Health | Participate in a guided mindful activity geared toward food appreciation. | Guided gratitude practice focused on food, health, and the body; final mindful-eating reflection | 5:37 |

**Note.** D2L = Desire2Learn; MYH = Mind Your Heart. The mindful-eating curriculum shown here was provided **in addition to** the DASH nutrition curriculum described in Multimedia Appendix A. Duration reflects the duration of recorded instructional or guided-practice content and does not include time spent reading module materials, completing reflective activities, setting behavioral goals, or practicing mindful-eating skills independently. Because the intervention was asynchronous and included self-directed practice, total time spent engaging with each module varied by participant. Modules were released weekly and previously released content remained accessible through the end of the six-week intervention.
