# Supplementary material for: Mind Your Heart—A Web-Based Dietary Approaches to Stop Hypertension Diet and Mindful Eating Intervention for Working Adults at Elevated Cardiovascular Risk: Pilot Randomized Controlled Trial
Source: JMIR Form Res. 2026 Oct 28;10:e106388. doi: 10.2196/106388 (PMC13716348; doi:10.2196/106388)
Supplement: Supplementary file 6 [file formative-v10-e106388-s005.docx]

**Multimedia Appendix 4**: Intervention Delivery, Planned Exposure, and Actual Exposure

This appendix summarizes intervention delivery procedures, planned participant exposure according to the study protocol, actual participant engagement with the intervention platform, and completion of study assessments.

**Part A**. Intended (Planned) Exposure

| **Intervention Component** | **Diet Only** | **MYH** |
| --- | --- | --- |
| DASH educational modules | 6 | 6 |
| Mindful eating modules | — | 6 |
| Total educational modules | 6 | 12 |
| Module release schedule | 1 module/week for 6 weeks | 2 modules/week (1 DASH, 1 mindful eating) for 6 weeks |
| Modules remained available after release | Yes | Yes |
| Weekly reminder emails | Up to 3/week | Up to 3/week |
| EMA prompts | 18 (3/week × 6 weeks) | 18 (3/week × 6 weeks) |
| Human support | Orientation, enrollment visit, outcome assessments; no individualized coaching | Orientation, enrollment visit, outcome assessments; no individualized coaching |
| Expected curriculum completion | 100% | 100% |

**Part B.** Actual Exposure

| **Measure** | **Diet Only** | **MYH** | **Overall** |
| --- | --- | --- | --- |
| Participants completing follow-up | 17/18 | 17/19 | 34/37 |
| Curriculum completed, %, mean (SD) | 90.5 (13.1) | 81.4 (26.8) | 85.9 (21.4) |
| Days logged into D2L, mean (SD) | 6.67 (1.94) | 5.78 (2.91) | 6.22 (2.33) |
| Time spent in D2L (minutes), mean (SD) | 238.7 (174.2) | 352.2 (169.2) | 295.4 (224.9) |
| Mean EMAs completed (SD) | 14.8 (3.2) | 13.5 (4.9) | 13.94 (4.07) |
| EMA completion, %, mean (SD) | 79.9 (17.2) | 75.0 (27.2) | 77.5 (22.6) |

**Part C.** Feasibility

| **Feasibility Metric** | **Diet Only**  **n (%)** | **MYH**  **n (%)** | **Overall**  **n (%)** |
| --- | --- | --- | --- |
| Completed postintervention follow-up | 17 (94.4) | 17 (89.5) | 34 (91.9) |
| Completed pre- and postintervention questionnaires | 17 (94.4) | 17 (89.5) | 34 (91.9) |
| Completed pre- and postintervention anthropometrics | 17 (94.4) | 17 (89.5) | 34 (91.9) |
| Completed pre- and postintervention blood pressure assessments | 17 (94.4) | 17 (89.5) | 34 (91.9) |
| Completed pre- and postintervention diet records | 15 (83.3) | 15 (78.9) | 30 (81.1) |
| Completed pre- and postintervention laboratory assessments | 15 (83.3) | 15 (78.9) | 30 (81.1) |
| Completed ≥50% of EMA prompts | 16 (94.1) | 16 (84.2) | 32 (88.9) |
| Completed ≥60% of assigned curriculum† | 17 (100.0) | 14 (73.7) | 31 (86.1) |
| Completed all study procedures (composite outcome) | — | — | 23 (67.6) |

**Note.** Percentages for postintervention follow-up and paired questionnaire, anthropometric, blood pressure, dietary, and laboratory assessments are based on all randomized participants (Diet Only, n=18; MYH, n=19; N=37). Percentages for EMA and curriculum completion are based on the 36 participants who remained enrolled during the intervention (Diet Only, n=17; MYH, n=19; N=36). “Completed all study procedures” represents participants who completed all required intervention activities and study assessments and is reported only as an overall composite feasibility outcome.

†Assigned curriculum refers to the intervention content assigned to each study condition (DASH curriculum for the Diet Only group and DASH plus mindful eating curriculum for the MYH group).

D2L=Desire2Learn learning management system; EMA=ecological momentary assessment. Engagement time represents D2L-generated cumulative time within intervention modules; periods of inactivity while content remained open could not be distinguished from active engagement.
