# Supplementary material for: Mind Your Heart—A Web-Based Dietary Approaches to Stop Hypertension Diet and Mindful Eating Intervention for Working Adults at Elevated Cardiovascular Risk: Pilot Randomized Controlled Trial
Source: JMIR Form Res. 2026 Oct 28;10:e106388. doi: 10.2196/106388 (PMC13716348; doi:10.2196/106388)
Supplement: Supplementary file 7 [file formative-v10-e106388-s006.docx]

**Multimedia Appendix 5**: Ecological Momentary Assessment (EMA) Engagement and Meal Characteristics

| **Measure** | **n/M** | **%/SD** |  |  |  |  |
| --- | --- | --- | --- | --- | --- | --- |
| Participants included | 36 |  |  |  |  |  |
| Scheduled EMA prompts | 648 |  |  |  |  |  |
| Completed EMA prompts | 502 | 77.5% |  |  |  |  |
| EMAs completed per participant, M (SD) | 13.94 | 4.07 |  |  |  |  |
| Participants completing ≥12 EMAs, n (%) | 31 | 86.1% |  |  |  |  |
| Average response time, M(SD) | 2:50:58 | 6:36:47 |  |  |  |  |
|  | **Overall n** | | **Diet Only** | | **MYH** | |
| **Feeling** | **n** | **%** | **n** | **%** | **n** | **%** |
| Relaxed | 356 | 68.7 | 190 | 71.7 | 166 | 65.6 |
| Rushed | 72 | 13.9 | 32 | 12.1 | 40 | 15.8 |
| Distracted | 54 | 10.4 | 21 | 7.9 | 33 | 13.0 |
| Stressed | 18 | 3.5 | 14 | 5.3 | 4 | 1.6 |
| Other | 18 | 3.5 | 8 | 3.0 | 7 | 2.8 |
| **Location** | **n** | **%** | **n** | **%** | **n** | **%** |
| Home | 215 | 43.1 | 124 | 48.4 | 91 | 37.4 |
| Work/Desk | 176 | 35.3 | 82 | 32 | 94 | 38.7 |
| Restaurant/Dining Hall | 44 | 8.8 | 28 | 10.9 | 16 | 6.6 |
| Outside/Picnic | 25 | 5.0 | 9 | 3.5 | 16 | 6.6 |
| Car/Public Transit | 23 | 4.6 | 11 | 4.3 | 12 | 4.9 |
| Other | 16 | 3.2 | 2 | 0.8 | 14 | 5.8 |
| **Meal Type** | **n** | **%** | **n** | **%** | **n** | **%** |
| Breakfast | 105 | 20.3 | 58 | 21.9 | 46 | 18.2 |
| Lunch | 290 | 56.0 | 145 | 54.7 | 145 | 57.3 |
| Dinner | 75 | 14.5 | 39 | 14.7 | 36 | 14.2 |
| Snack | 42 | 8.1 | 19 | 7.2 | 23 | 9.1 |
|  | ***M*** | ***SD*** | ***M*** | ***SD*** | ***M*** | ***SD*** |
| **Perceived Meal Healthfulness** | 4.62 | 1.6 | 4.45 | 1.65 | 4.8 | 1.52 |

**Note.** EMA = ecological momentary assessment. Participants received three EMA prompts per week for six weeks (18 scheduled prompts). Percentages for meal type, feelings during meals, and eating location are based on non-missing responses.
